# Optimized Seizure Detection Algorithm: A Fast Approach for Onset of Epileptic in EEG Signals Using GT Discriminant Analysis and K-NN Classifier

**Published:** 2016-06-01

**Authors:** Kh. Rezaee, E. Azizi, J. Haddadnia

**Affiliations:** 1 Hakim Sabzevari University of Sabzevar, Department of Electrical and Computer Engineering, Sabzevar, Iran; 2 Sabzevar University of Medical Science, Department of Medical Physics and Biomedical Engineering, New Technologies Research Center, Sabzevar, Iran

**Keywords:** EEG Signals, Epileptic Seizure, General Tensor Discriminant Analysis (GTDA), K-NN, Wavelet Transform

## Abstract

**Background:**

Epilepsy is a severe disorder of the central nervous system that predisposes the person to recurrent seizures. Fifty million people worldwide suffer from epilepsy; after Alzheimer’s and stroke, it is the third widespread nervous disorder.

**Objective:**

In this paper, an algorithm to detect the onset of epileptic seizures based on the analysis of brain electrical signals (EEG) has been proposed. 844 hours of EEG were recorded form 23 pediatric patients consecutively with 163 occurrences of seizures. Signals had been collected from Children’s Hospital Boston with a sampling frequency of 256 Hz through 18 channels in order to assess epilepsy surgery. By selecting effective features from seizure and non-seizure signals of each individual and putting them into two categories, the proposed algorithm detects the onset of seizures quickly and with high sensitivity.

**Method:**

In this algorithm, L-sec epochs of signals are displayed in form of a third-order tensor in spatial, spectral and temporal spaces by applying wavelet transform. Then, after applying general tensor discriminant analysis (GTDA) on tensors and calculating mapping matrix, feature vectors are extracted. GTDA increases the sensitivity of the algorithm by storing data without deleting them. Finally, K-Nearest neighbors (KNN) is used to classify the selected features.

**Results:**

The results of simulating algorithm on algorithm standard dataset shows that the algorithm is capable of detecting 98 percent of seizures with an average delay of 4.7 seconds and the average error rate detection of three errors in 24 hours.

**Conclusion:**

Today, the lack of an automated system to detect or predict the seizure onset is strongly felt.

## Introduction

Epileptic seizures are temporary periods entangling hyperactivity and hyper-synchronization of a great number of neurons within one or more neural networks producing disruptive symptoms[1]. Epilepsy may be the result of a mutation in molecular mechanism that regulates neural behavior, transfer and organization. It may be caused by brain injuries such as a severe head trauma, stroke, brain infection or a malignant brain tumor[2]. Fifty million people worldwide suffer from epilepsy; after Alzheimer’s and stroke, it is the third widespread nervous disorder[3]. From a surgical perspective, severe seizures restrict independent and social activities of the person with consequences like seclusion and economic problems. The most critical consequences of seizures concern the possibility of experiencing burn, rupture and breakage of skull as well as increased chance of unexpected events such as death. Surgery of hard seizures requires identifying the seizure focus, i.e. where seizure begins. To determine the location of seizure, electrical brain signals (EEG), brain imaging by MRI and functional brain imaging during seizure and non-seizure fits by SPECT can be used[4]. The onset of seizure is usually detected by EEG analysis. Electroencephalography (EEG) is a multi-channel recording of electrical activity produced by a set of neurons within the brain. The channels reflect internal activity of different brain areas. In this method, measurement is taken by installing non-invasive electrodes on scalp. EEG features of scalp and intracranial areas among people with epilepsy are changeable in both epileptic and non-epileptic states[5]. To detect the onset of a seizure, a set of EEG channels generate a rhythmic activity that is the result of over-harmonizing activity of the brain. The position of the channels involved and the content of the rhythmic activity vary from person to person.

In addition, EEG effects of a patient’s seizure may be similar to the effect of a non-seizure disorder of the same patient or another[6]. EEG signals are classified with respect to their frequency components. In other words, they are classified into delta (f < 4Hz), theta (4 < f < 8Hz), alpha (8 < f < 12Hz), beta (12 < f < 30Hz) and gamma (f > 30Hz)[7].

A seizure detector can be grouped as a seizure onset detector or as a seizure accident detector[8]. The goal of a seizure onset detector is to distinguish when a seizure has started with the least possible delay, but not necessarily with the greatest possible accuracy. In contrast, the goal of a seizure accident detector is to recognize seizures with the highest possible accuracy, but not necessarily with the least delay[9, 10]. Seizure onset detectors are employed for applications requiring a response to a seizure, while seizure accident detectors are used for applications requiring an accurate account of seizure activity over a period of time. In seizure onset detectors, quick initiation is serious, because the accuracy of such imaging studies diminishes the delay between seizure onset and infusion of the imaging radiotracer greatly[11]. Within the realm of therapy, seizure onset detection could be used to trigger neuro-stimulators designed to affect the progression of a seizure. In this application, rapid initiation is serious, because the likelihood of affecting a seizure seems to decrease longer delay between the onset of a seizure and the start of stimulation[12]. Finally, within the realm of warning, seizure onset detection could help a patient or care-provider to ensure safety or administer a fast-acting anticonvulsant. The spectral energy redistribution is caused by hyper-synchrony of neurons within an epileptic neural network and consists of an appearance or disappearance of frequency components within 0-25 HZ band[13, 14]. However, what spectral components vanish or rise to prominence varies among patients. Furthermore, EEG channels demonstrating spectral energy change also varies among patients since it is a function of the cerebral site of origin of a seizure.

### Related Works

One of the simplest detectors of seizure onset was developed by Gotman[15]. Gotman’s algorithm, tracked harmonic rhythmic activities in EEG signals as a symptom of seizure. The algorithm searched a number of EEG channels for the presence of a rhythmic activity with a beat frequency of 3-30HZ and the amplitude less than three times of the largest window. Whenever the rhythm exceeded a threshold and lasted for about 4 seconds, the seizure detection was alarmed. Gotman’s algorithm could detect seizures with rhythmic activity below 20Hz; the Gotman algorithm is not successful in recognizing seizures consisting of EEG containing a mixture of frequencies or those with low amplitude high frequency activity. However, it was not able to detect seizures with a combination of frequencies or seizures with low amplitude and high frequency. Reveal Seizure Detection was developed by Wilson[16]. Reveal Algorithm analyzed 2-sec epoch of each channel into time-frequency components, using neural network rules to determine whether features extracted from the components of a channel were in accordance with a seizure occurrence. As reported by Wilson in[16], Reveal algorithms detected 76% of the 672-recorded cases of epileptic seizure in 425 patients, showing an error detection rate of 0.11 for patients without epilepsy. When Reveal algorithm was designed specifically for each patient[16-18], detection accuracy improved from 0.62 to 0.34 error per hour, and a sensitivity of 78% was achieved. Meier proposed a general seizure onset detector, though he failed to offer one specifically for patients[19]. Shoeb proposed a seizure onset detector system specific to each patient[20-22]. His algorithms used feature extraction in the frequency band of 0-25 HZ by a filter bank of 3HZ and SVM classification to categorize the features extracted into seizure and non-seizure classes. His algorithm was tested on 844 hours of EEG signals taken from scalp of over 23 pediatric patients. The algorithm detected 96% of 163 seizures with an average delay rate of 4.5 seconds and the average error of 0.07 per hour. Shoeb’s algorithm has higher sensitivity than Gotman algorithm, but non-rhythmic activity seizure had been recognized with large latency.

In this algorithm, GTDA was used to select effective features from seizure and non-seizure EEG signals. In next step, we evaluated our detector based on three metrics: latency, specificity and sensitivity. We compared our detector with other works that had been presented in the literature. In comparison with other works, our proposed algorithm is able to reduce latency and can be used to initiate just-in-time therapy methods such as VNS and TNS. This proposed algorithm was tested on 91 seizures and 1360 hours of non-seizure taken from 57 patients. The algorithm was able to detect 96% of seizures with an average delay rate of 1.6 sec and error detection of 0.45% per hour. His approach depended on whether the tested seizure was in one of the six defined categories. This article presents a seizure onset detection algorithms specific to the patients aiming at reducing the response period of detector by selecting effective features of the EEG signals. 

## Material And Methods

In this paper, we propose an online EEG-based epileptic seizure onset detection algorithm based on effective features selected by applying general tensor discriminant analysis and K-NN classifier. Next section describes general structure of the proposed algorithm. 

**Figure 1:**
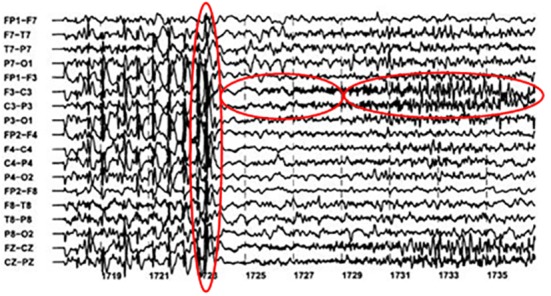
An Example of Seizure in EEG Signals of Patient A

**Figure 2:**
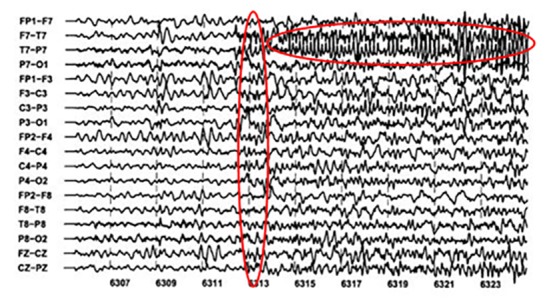
An Example of Seizure in EEG Signals of Patient B

Figure 1 and figure 2 show seizures of patients A and B, respectively. In figure 1, seizure began in the second 1723, which included the flattening of the EEG signals across channels as detected by the emergence of a beta rhythm band on channels (F3-C3, C3-P3). Then, it lasted for a few seconds and the amplitude increased with its frequency falling in theta band.

**Figure 3:**
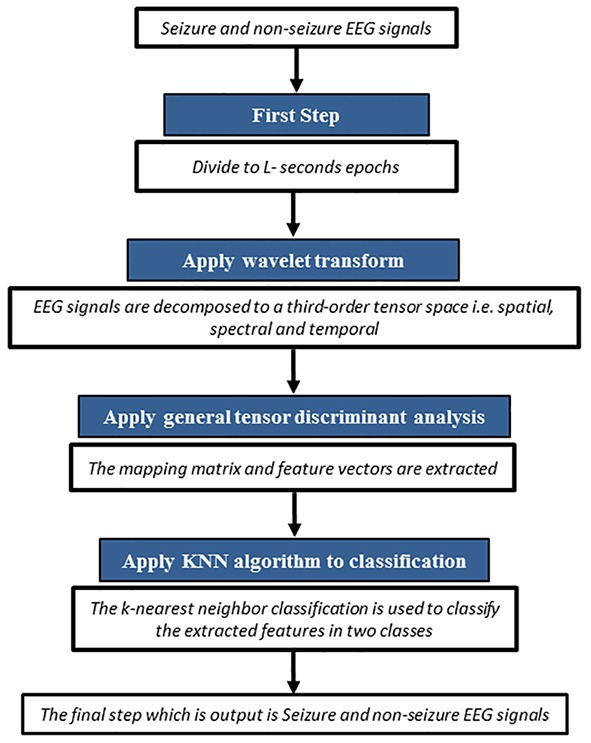
Implementation of Proposed Algorithm

In figure 2, seizures began in the second 6313, continuing with a prominent rhythm in theta band on channels (F7-T7, T7-P7). Other EEG channels registered changes too. For example, channel (C3-P3) developed a theta band rhythm. The overall structure of proposed EEG-based seizure onset detection algorithm is shown in figure 3.

The dataset used to evaluate the performance of patient-specific detector operator included consecutive EEG recordings from 23 pediatric patients (younger than 18). Signals which were measured to assess epilepsy surgery, were collected from Children’s Hospital Boston. The sample frequency vector of 256 Hz was obtained by 18 channels. In general, 844 hours of consecutive EEG was recorded from 23 patients, containing 163 occurrences of seizures. EEG datasets were separated into hourly intervals. The data without seizure occurrence were labeled as non-seizure class and data containing seizure occurrences were labeled as seizure class. EEG datasets were separated into hourly intervals. The data without seizure occurrence were labeled as non-seizure class and data containing seizure occurrences were labeled as seizure class. The details of these datasets have been shown in Table 1[23].

**Table 1:** EEG Data Specifications

**patient**	**sex**	**age**	**seizure type**	**origin**	**No. seizures**	**non-seizures (hours)**	**seizure (min)**
1	F	11	SP.CP.GTC	Temporal	6	46	7.36
2	M	11	SP.CP	Frontal	3	29	2.86
3	F	14	SP.CP.GTC	Temporal	7	32	7.46
4	M	22	SP.CP.GTC	Temporal, Occipital	4	93	6.3
5	F	7	SP.CP	Frontal	4	35	9.3
6	F	1.5	SP.CP.GTC	Temporal	7	54	2.53
7	F	14.5	SP.CP.GTC	Temporal	3	61	5.41
8	M	3.5	SP.CP.GTC	Temporal	5	20	15.33
9	F	10	SP.CP	Frontal	4	65	4.6
10	M	3	SP.CP.GTC	Temporal	7	45	7.45
11	F	12	SP.CP	Frontal	3	31	13.43
12	F	2	SP.CP	Frontal	24	22	39.26
13	F	3	CP.GTC	Temporal, Occipital	12	34	9.58
14	F	9	SP.CP.GTC	Temporal	8	26	2.81
15	M	16	CP.GTC	Frontal, Temporal	20	37	33.91
16	F	7	SP.CP.GTC	Temporal	10	19	1.41
17	F	12	SP.CP.GTC	Temporal	3	24	4.88
18	F	18	CP.GTC	Temporal, Occipital	5	31	5.28
19	F	19	SP.CP	Frontal	3	26	3.93
20	F	6	SP.CP.GTC	Temporal	8	27	4.9
21	F	13	SP.CP.GTC	Temporal	4	23	3.31
22	F	9	CP.GTC	Temporal, Occipital	5	28	3.4
23	F	6	SP.CP	Frontal	8	36	7.06
				Total	163	844	201.76

### Spectral and Spatial Feature Extraction

Given the non-static nature of EEG signals, wavelet transform can be a powerful tool to divide signals into several sub-bands or to extract appropriate characteristic features[13, 24]. Spectral features can be used to establish the difference between the two classes. In the proposed algorithm, discrete wavelet transform has been used as a powerful means to divide EEG signals and to present them in spectral, spatial and temporal amplitudes. For a sample epoch *X_(c,t)_* in channel c and time t, the wavelet coefficients (sub-bands) and *x_(c,f,t)_* can be achieved by its convolution and a mother wavelet *H_(t,f)_* according to the equation (1):

$$x_{\left(c,f,t\right)}=\left\|H_{\left(t,f\right)}*X_{\left(c,f\right)}\right\|\;\;(1)$$

The mother wavelet Morlet (Equation 2) was used as a mother wavelet to calculate coefficients in M sub-bands as this wavelet showed an acceptable performance in a variety of EEG signals applications[25]. Ω is the center frequency and   is the bandwidth parameters. Here, we assumed Ω = 1 and σ = 2.

$$\Psi \left(t\right)=\frac{1}{\sqrt{\pi \sigma }}\exp\left(2i\pi \Omega t\right)\exp\left(-\frac{t^{2}}{\sigma }\right)\;(2)$$

**Figure 4:**
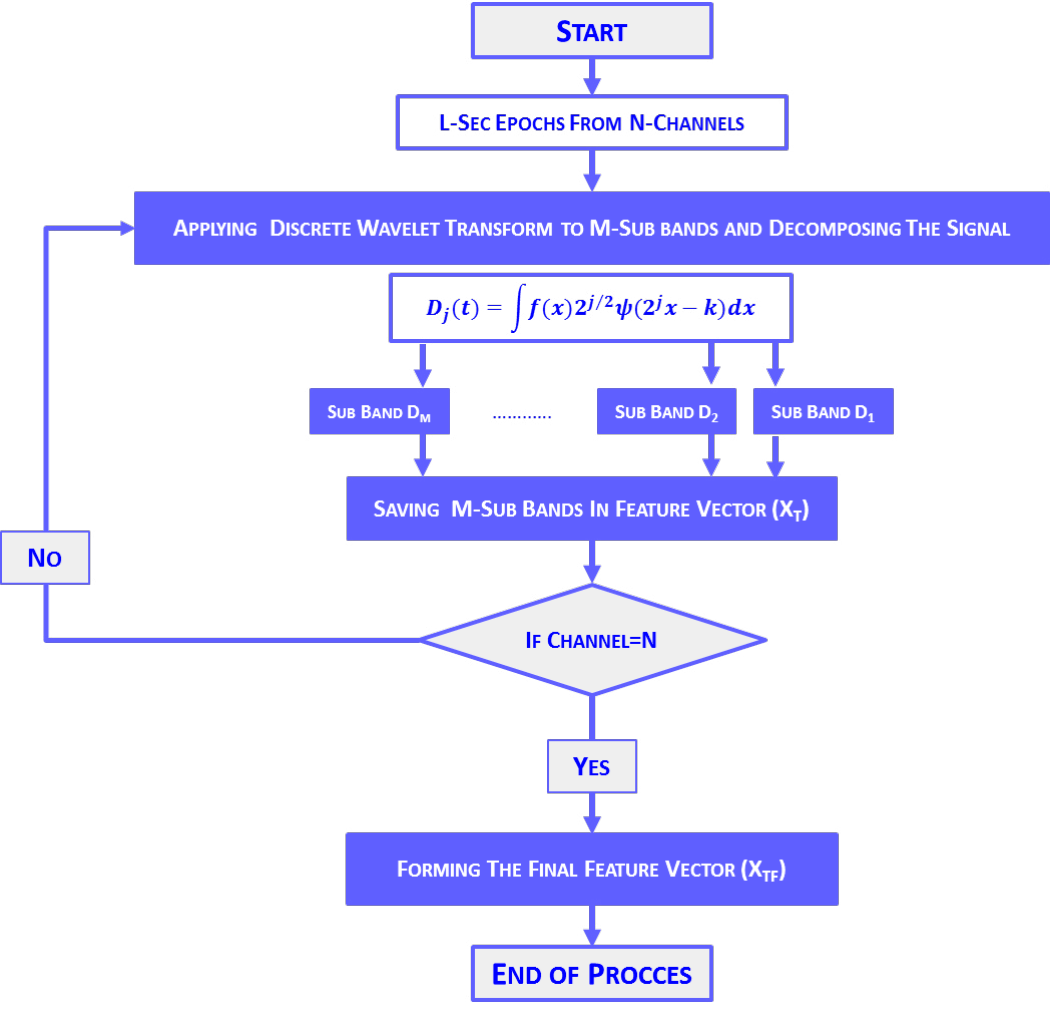
How Spectral and Spatial Features are Extracted in Proposed Algorithm

In addition, EEG channels involved in rhythmic seizure activities can be used as an indicator for detecting seizures. Figure 4 shows how spectral and spatial feature are extracted.

The extraction of spectral features can be used for all channels. To consider spectral and spatial data for an L-sec period in time t (t=T), the extracted features are linked to develop a composite feature vector *X_T_* with *N*×*M* elements (N is the number of channels), and the relationship between spatial structures of different channels will be established automatically.

### Feature Reduction Based on General Tensor Discriminant Analysis (GTDA) Technique

Many features extracted from EEG signals representing seizure or non-seizure do not have any marked characteristic, thus increasing the calculations costs in classifications. General tensor discriminant analysis (GTDA)[26] can be used as a useful method to reduce the dimension of feature vectors, which is able to save the information extracted from EEG signals without deleting minor data. Tensors are multi-dimensional arrays of values which transform linearly under coordinate transformations[27, 28]. If $X\in R^{N_{1}\times N_{2}\times \cdots \times N_{M}}$ then M is the order of a tensor, that is denoted by $X_{n_{1},n_{2},\cdots ,n_{M}}$ High order tensors are traditionally scanned into vectors (vectorization) by techniques such as PCA and MSA. But during vectorization, a great deal of useful structure information is lost. On the other hand, when the resulting vector is much larger than the number of examples in the training set, USP is accrued. GTDA can be used to preserve discriminant information in the original data and reduce USP. GTDA algorithm[29] gives a number of training samples x_i,j_ ϵ R^N^ in c classes, where i is the class number, 1 ≤ i ≤ c and j is the sample index in the j^th^ class, 1 ≤ j ≤ n, the goal of GTDA is to find a projection of the x_i,j_ , which is optimal for separating different classes in a high dimensional space. In this strategy, the projection matrixU*, which is defined by a set of vectors U = [u_1_, u_2_, …, u_c-1_] is chosen to maximize the ratio between the trace of Sb and the trace of S_W_.

U*=max⁡tr(UTSbU-ξtr(UTSWU))  (3)UTU=1arg

Where *S_b _, S_W_* , are between-class scatter matrix and within-class scatter matrix, respectively defined as (4),(5):

$$S_{b}=\frac{1}{n}\sum_{i=1}^{c}n_{i}\left(m_{i}-m\right){\left(m_{i}-m\right)}^{T}\;\;(4)$$

$$S_{w}=\frac{1}{n}\sum_{i=1}^{c}\sum_{j=1}^{n_{i}}(x_{i.j}-m_{i}){\left(x_{i.j}-m_{i}\right)}^{T}\;\;(5)$$

Where

$$n=\sum_{i=1}^{c}n_{i}.m_{i}=(\frac{1}{n_{i}})\sum_{j=1}^{n_{i}}x_{i.j\;\;}\;(6)$$

$$m=\left(\frac{1}{n}\right)\sum_{i-1}^{c}\sum_{j-1}^{n_{i}}x_{i.j}\;(7)$$

In Eq. (3), $U\epsilon R^{N\times N^{*}}$ (N* ≪ N) constrained by UT = I, is the projection matrix and ζ is a tuning parameter. To extract N*features simultaneously, ζ is estimated as $\sum_{i=1}^{N^{*}}\lambda_{i}$ , where ${\left.\lambda_{I}\right|}_{i=1}^{l}$ are the largest N* eigenvalues of $S_{W}^{-1}S_{B}$.

In real applications, suitable ζ cannot be manually determinate. In this paper, we automatically select ζ during training procedure. An accurate solution of Eq. (3) can be obtained by the alternating that has been described in[30, 31]. table 2 shows key steps to find the l^th^ projection optimization matrix $U_{l}^{\left(t\right)}$ in t^th^ iteration.

**Table 2:** Alternating procedure to find optimal projection matrix

Begin ${Set\;U}_{L=1}^{\left(0\right)}\left|\begin{array}{c} M\\ l-1 \end{array}=1_{N_{l}\times N_{L}}\left(All\;entries\;of\;U_{l}^{\left(0\right)}are\;1\right)\right.$ For t=1 to Err (t) < ε For l=1 to l=M $B_{l}^{\left(t-1\right)}=\sum_{i=1}^{c}\left[n_{i}{mat}_{l}((M_{i}-M){\bar{\times }}_{l}(U_{l}^{\left(t-1\right)}{)}^{T}\right)$ ${mat}_{l}^{T}(\left(M_{i}-M\right){\bar{\times }}_{l}\left(U_{l}^{\left(t-1\right)}{)}^{T}\right)]$ $W_{l}^{\left(t-1\right)}=\sum_{i=1}^{c}\sum_{j=1}^{n_{i}}[{mat}_{l}((M_{i.j}-M_{i}){\bar{\times }}_{l}(U_{l}^{\left(t-1\right)}{)}^{T})$ ${mat}_{l}^{T}(\left(x_{i.j}-M_{i}\right){\bar{\times }}_{l}\left(U_{l}^{\left(t-1\right)}{)}^{T}\right)]$ Ul*(t)=max⁡tr(UTBlt-1-ξWlt-1U)Uarg End of for $Err\left(t\right)=\sum_{l=1}^{M}\left\|\left|U_{l}^{\left(t\right)}(U_{l}^{\left(t-1\right)}{)}^{T}\right|-1\right\|\leq \epsilon$ End of for Optimization matrix $U_{l}^{\left(t\right)}$ End

In table 2, ${\left.X_{i.j}\right|}_{1<i<c}^{1<j<n_{i}}\in R^{N_{1}\times N_{2}\times \cdots \times N_{M}}$ are training tensors. The projected tensor $Y_{i.j}\in R^{N_{1}\times N_{2}\times \cdots \times N_{M}}$ to replace the original general tensor X for recognition is calculated as (8): $Y=X\prod_{l=1}^{M}U_{1}\;\;\;(8)$ To determine the tuning parameter ξ automatically, we adjust ξ in the t^th^ training iteration and the l^th^ order by setting ξ(1) equal to the maximum eigenvalue of ${\left(W_{l}^{\left(t-1\right)}\right)}^{-1}B_{l}^{\left(t-1\right)}$ .

### Classification of Selected Features Based on Adaptive KNN Algorithm

One of the supervised algorithms that are commonly used for monitoring different states of input data is K-nearest neighbor algorithm (K-NN), which classifies input data based on the number of clusters N. 

This algorithm is based on the minimum distance between data and default centers using different norms to calculate the minimum value. After identifying K near neighbors, groups that can establish the nearest neighbors are investigated. In the proposed method, KNN algorithm specifies the tested sample class in three steps: 

• The distance between the tested sample and the training samples in all classes is calculated based on one of the Euclidean metrics, i.e. Manhattan or City Block. 

• The nearest training sample relative to the test sample will be determined based on the K^th^ neighboring distance.

• For each class, the number of training samples in K^th^ neighboring distance is determined and the class with the most training samples in that neighboring distance is introduced as the test class.

In Table 3, Structural and Procedure Conditions of K-NN are Shown.

**Table 3:** Proposed Adaptive K-NN with Training Border

Begin Initialize j ← 0, D ← data set, and n ← #prototypes Construct the full Voronoi diagram of D Do j ← j + 1; for each prototype x’j Find the Voronoi neighbors of x’j If any neighbor is not from the same class as, Then Mark x’j Until j = n Discard all points Construct the Voronoi Prototypes End

## Experimental Results

By applying GTDA technique, prominent features are selected from extracted feature, and finally the selected feature will be categorized by KNN classification. After extracting selected features by GTDA, the max difference between seizure and non-seizure signals is generated, producing a dramatic increase in detection sensitivity. Overall, proposed algorithm has the following properties:

• Increased accuracy of performance

• Negligible average error 

• Reduced user intervention

• Suitable for starting vagus nerve stimulator

### Performance Evaluation

The accuracy of a classification system is frequently evaluated by means of three measures: latency, sensitivity and specificity.

#### Response Period or Delayed Detection (D)

It is the delay between the start of epilepsy marked by EEG technician and the detection of seizure activity by the detector.

#### Latency

The electrographic onset of a seizure refers to the onset of scalp EEG changes associated with a seizure. The delay between the expert-marked seizure onsets within the EEG detector declaration of seizure activity is known as latency.

#### Sensitivity

The percentage of seizure correctly identified which is obtained by (9):

$$Sensitivity=\frac{Correctly\;detected\;positives}{Total\;actual\;positives}=\frac{TP}{TP+FN}\;\;(9)$$

#### Specificity

The specificity refers to the number of times, over the course of an hour, a detector declares the onset of seizure activity in the absence of an actual seizure which is calculated by (10):

$$Specificity=\frac{Correctly\;detected\;negatives}{Total\;actual\;negatives}=\frac{TN}{TN+FP}\;\;(10)$$

## Results of Simulation

The proposed algorithm is implemented by MATLAB software to evaluate the performance. To calculate latency, sensitivity and specificity of the proposed algorithm, the detector is trained on each patient’s EEG data, separately. Adaptive K-NN classifier used the city block distance and K=10. To calculate the response period, sensitivity and accuracy of the proposed algorithm, the detector was trained on EEG data of each patient separately. To classify KNN, the distance measure of City Block with the target value of 13 was selected. The parameters influencing the performance of the detector were set according to Table 4.

**Table 4:** Values Presumed for Trained Classifier Parameters

**PARAMETERS**	**DESCRIPTION**	**INITIALIZING**
2 seconds	EEG epoch length	2 seconds
18 channels	Number of EEG channels	18 channels
5 sub-band	Number of the obtained sub-band by applying wavelet transform	5 sub-band
20-24 hours	Number of the used hours to extract training non-seizure vectors	20-24 hours
20 seconds	Number of seconds into seizure used to extract training seizure vectors	20 seconds
3-10 seizure	Number of seizures used to extract training seizure vector	3-10 seizure

**Figure 5:**
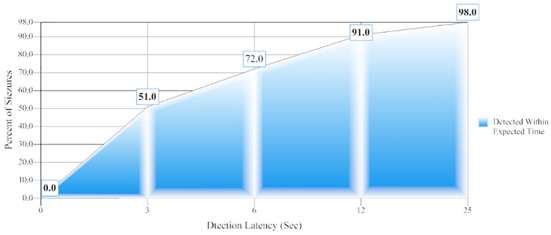
Percentage of Detected Seizures within a Specified Latency

**Figure 6:**
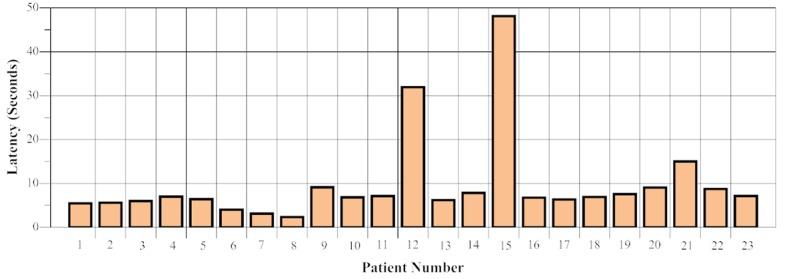
Percentage of Detected Seizures within a Specified Latency

Figure 5 shows the percentage of test seizures detected within a specified latency. Moreover, 51% of 163 test seizures were detected with a 3-second delay, 72% with a 5-second delay and 91% with a 10-second delay. Figure 6 displays the average detector response period for each of the 23 patients. For the majority of patients, most seizures were detected with a response period of 4.7 seconds.

**Figure 7:**
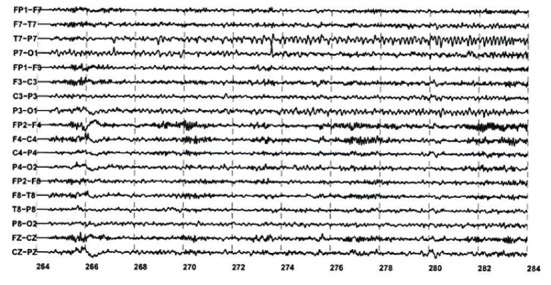
Example of an EEG Seizure in Patient No. 15. Seizure Began in the Second 272 with a Theta-Band Rhythm, which is most Prominently Observable on Channels T7-P7

**Figure 8:**
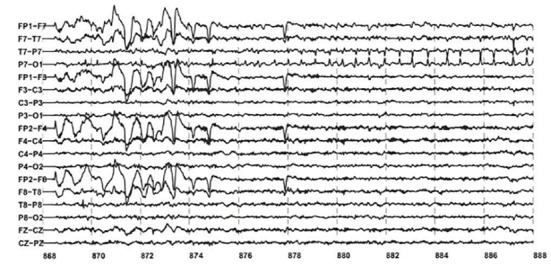
Another Example of EEG Seizures of Patient No. 15. These Seizures Began in the Second 876 with a Training of Pulses on Channel P7-O1.

One of the seizures was detected with 50-second delay for patient No. 15. This long response period is because epileptic EEG seizure was different from spatial features of training seizures.  Figure 7 shows a training seizure for the same patient that began in second 272 and continued with a rhythmic theta-band on channels T7-P7. Figure 8 shows the tested seizure that occurred for patient No. 15 with detection error. It began from the second 876 and included a train of pulses on channel P7-O1. The detector had problem detecting the onset of this seizures because the specter and its amplitude differed from training seizures. The training seizures are similar to the ones shown in figure 7.

### Sensitivity Estimation

**Figure 9:**
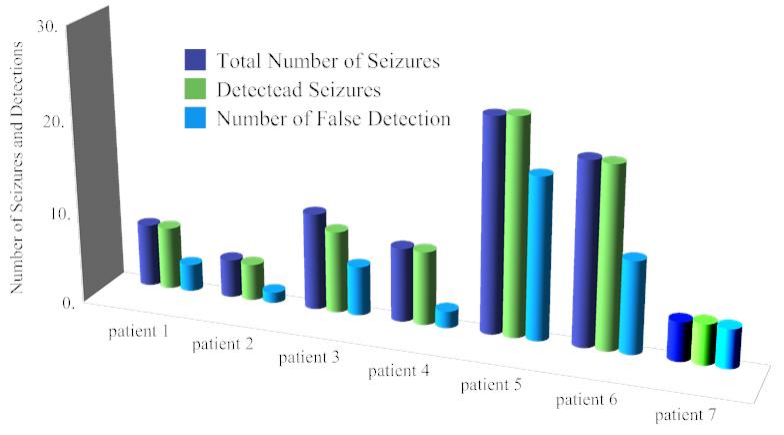
Sensitivity of Patient-Specific Seizure Detector. Overall, 98% of 163 Seizures were Revealed.

Figure 9 shows the sensitivity of patient-specific algorithm for 7 patients from 23 patients. More than 98% of 163 seizures were detected correctly.

### Accuracy Estimation

**Figure 10:**
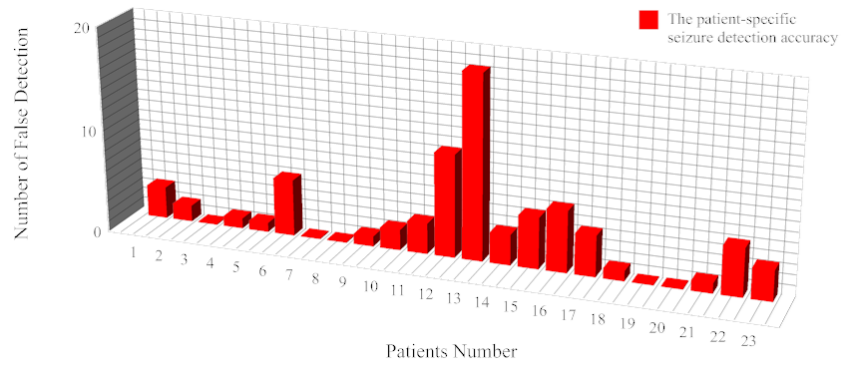
Sensitivity of Patient-Specific Seizure Detector. Overall, 98% of 163 Seizures were Revealed.

Figure 10 shows the number of detection errors made by the detector for 23 patients in 24 hours. For most patients (18 patients), the detector made between 0 and 5 errors in 24 hours. For example, for patient No. 13, the algorithm had 18 detection errors in 24 hours. This great detection error can be explained in terms of the short-term breaks of rhythmic brain activity, which are similar to the onset of epileptic activity in terms of spatial and spectral features. Ti improves the accuracy of detection algorithm for patient No. 13, the detection can be declared only when seizures continue for a long time leading to a rise in the response period.

## Discussion

Overall, 98% of 163 test seizures were detected. For most patients (18 of 23), the detector declares between 0 and 5 false detentions per 24 hours. For patient 13, the algorithm declares 20 false detections per a 24-hour period. The reason for the high false alarm rate is the presence of short bursts of rhythmic activity that match seizure onset signature both in spectral and spatial character. In these experiments, a number of sub-bands (M) were used to generate feature vector. Increasing the number of sub-bands does not greatly impact the detection latency or the sensitivity of the detector. The average, across 23 subjects, latency of detectors with 3, 5 and 7 sub-band was 4.5, 4.8 and 5 seconds, respectively. All detectors had a sensitivity of 98%. Increasing the number of sub-bands appears to improve the specificity of a detector. The average specificity of a detector that uses three sub-bands is greater than 8 false detections per 24 hours, and that of a detector with seven-sub bands is less than 4 false detections per 24 hours. 

Our detector uses effective features that are extracted by wavelet transform from spectral, spatial and temporal domain of EEG signals and selected by GTDA. Utilizing this strategy, the discriminative information in training tensors is preserved as a benefit in comparison with PCA and MSA. Shoeb’s detector extracted features from 0 to 25HZ frequency band by means of a 3HZ band width filter and a support vector machine (SVM) was used as classifier[20]. Our algorithm exhibits a shorter latency, a higher sensitivity, and a comparable specificity relative to Shoeb’s algorithm. High sensitivity increases the capability of detector to recognize seizures in order to initiate just-in-time therapy methods. Our detector can recognize more seizure with less delay in comparison with other algorithms. The increased number of sub-bands does not affect the response period and sensitivity of the detector. On average, for 23 patients, the response period of the detector using 3, 5 and 7 sub-bands was respectively 5.4, 4.8, and 5 seconds, and all detections had a sensitivity of 98%. Furthermore, it can be concluded that a rise in the number of sub-bands would have a significant impact on the accuracy of the detector. The average accuracy of detection requires three sub-bands with more than 8 errors, but even with seven sub-bands, achieving less than 4 errors in 24 hours is possible. The proposed algorithm uses wavelet analysis to extract features and GTDA to reduce the dimension of feature vector. It is while the Shoeb algorithm[21, 22] uses a 3HZ filter for feature extraction and SVM for classification of feature vectors.

Both algorithms were tested on the same dataset. The results suggest that the proposed algorithm has shorter response period, higher accuracy and sensitivity compared to algorithms such as Shoeb algorithm in detecting the onset of epileptic seizure. In Table 5, the comparison between the results of the proposed algorithm with Shoeb algorithm, which is one of the best algorithms in detecting the onset of epileptic seizure, has been shown.

**Table 5:** Comparison of Results Obtained from Proposed Algorithm and Shoeb Algorithm

**Proposed Algorithm**	**Accuracy**	**Sensitivity**	**Delayed Detection**
Our work	0.12 error in one hour	0.98	4.7 Sec
Shoeb	0.17 error in one hour	0.96	5.2 Sec

## Conclusion

This paper proposed an algorithm with acceptable sensitivity and accuracy for patient-specific seizure onset detection. First, L-sec periods from seizure and non-seizure signals were broken into several sub-bands using wavelet transform. Then, selected features were extracted by applying GTDA. At the end, the nearest neighbor classification was trained to classify feature vectors extracted from seizure and non-seizure signals, so that they could be used to determine the class of the training examples. Features selected by GTDA were able to establish the greatest distinction between seizure and non-seizure signal, and thereby increasing the sensitivity of the detector. The proposed detector was tested on 844 hours of EEG signals taken from the scalp of 23 pediatric patients. The algorithm detected 98% of 163 seizures with an average delay of 4.7 seconds and the average error of 0.12 errors per hour. The authors intend to find the value of K based on optimization tests such as a fuzzy system or evolutionary algorithms. Furthermore, enhancing feature vector and the detector of seizure stop are among future works of the author in order to improve the performance of the proposed algorithm. 

In several tests, we studied influence of important parameters of the proposed detector. Also, we compared our detector with other works that had been presented in the literature. In comparison with other works, our proposed algorithm is able to reduce the latency and can be used to initiate just-in-time therapy methods such as VNS and TNS.
